# Carbon emissions accounting and uncertainty analysis in campus settings: A case study of a university in Sichuan, China

**DOI:** 10.1371/journal.pone.0321216

**Published:** 2025-04-16

**Authors:** Chen Yang, Tong Yao, Dong Shiming, Wenjie Jiang

**Affiliations:** 1 Sichuan University Jinjiang College, Meishan, China; 2 Sichuan Tourism University, Cheng Du, Sichuan, China; 3 Sichuan University, Cheng Du, China; 4 School of Civil Engineering and Architecture, Southwest University of Science and Technology, Mianyang, China; University of Coimbra: Universidade de Coimbra, PORTUGAL

## Abstract

Within the context of advancing global sustainable development goals, universities are recognized as leaders in energy conservation and emissions reduction within the education sector. Universities should actively engage in the accounting and analysis of carbon emissions. This study uses Sichuan University Jinjiang College(Hereafter referred to as J University) in Sichuan, China, as a case study, where the campus’s carbon emissions for the year 2023 were calculated using the Emission Factor Method and the Delphi Method. The uncertainty associated with these emissions was further explored using Monte Carlo simulation. The results indicate that the net carbon emissions of J University amounted to 44,584.33 tons of CO_2_ equivalent (tCO_2_e), with per capita emissions of 1.89 tCO_2_e. The primary sources of campus carbon emissions, in descending order, include electricity (18879.94tCO_2_e), natural gas (8647.25tCO_2_e), business travel (5224.55tCO_2_e), campus commuting (3852.33tCO_2_e), food (3444.67tCO_2_e), and thermal energy (2566.63tCO_2_e). Among these sources, the carbon emissions from electricity, natural gas, and thermal energy were closely correlated with seasonal and regional factors. The uncertainties related to commuting and business travel had the most significant impact on the overall carbon emissions accounting for the campus. The study presents a framework for campus carbon emission accounting, providing a concrete case study for future researchers in this field. In particular, an in-depth exploration of statistical uncertainties is conducted, offering a scientific basis for the accurate calculation of carbon emissions in future studies.

## 1. Introduction

Global warming is measured by the concentration of greenhouse gases present in the atmosphere [1–3]. Carbon emissions constitute a significant component of these greenhouse gases. To effectively address the significant challenge of global warming, universities should proactively engage in carbon emissions assessment to contribute meaningfully to environmental protection [4,5].

According to the “WMO Greenhouse Gas Bulletin (2022), Issue 19,” published by the World Meteorological Organization (WMO) on November 15, 2023, the global annual mean atmospheric concentration of major greenhouse gases reached a new peak in 2022, with carbon dioxide (CO_2_) measured at 417.9 ± 0.2 ppm, representing 150% of pre-industrial (pre-1750) levels [6]. China,as one of the largest carbon emitters globally, is actively implementing measures to reduce carbon emissions and facilitate the transition to a low-carbon economy. Universities, as a vital component of society, are entrusted with roles in talent cultivation, scientific innovation, and social service, and are expected to play a pivotal role in this transformation [7]. As of 2022, China has 3,013 higher education institutions, with a total enrollment of 46.55 million students across these institutions nationwide [8]. Significant potential exists for emission reductions within Chinese higher education institutions [9,10].

## 2. Literature review

Universities have consistently responded to emissions reduction commitments, such as the Paris Agreement and the Kyoto Protocol. In 2019, more than 7,000 universities globally launched the “Climate Emergency” initiative, pledging to achieve carbon neutrality on their campuses. In 2024, the China Association of Building Energy Efficiency issued the “Guidelines for Campus Carbon Emission Accounting in Higher Education Institutions,” promoting the evaluation and accounting of university carbon footprints. Fatin Samara et al. employed greenhouse gas protocol methods to calculate carbon footprints, demonstrating that the primary contributors to campus CO_2_ emissions are electricity consumption and commuting within the university [11]. Betanti and Rahman evaluated the carbon footprint of Universities Pertamina in Indonesia using direct sampling, surveys, and carbon emission factor calculations, concluding that electricity is the predominant source of carbon emissions at the university [12]. Rubén Mendoza-Flores et al. assessed the greenhouse gas inventory of the Cuajimalpa campus of the Autonomous University of Mexico City and calculated the carbon footprint of these emissions. Their activity-based emission analysis revealed that, in 2016, 51% of the university’s total carbon emissions were attributed to commuting, 24% to electricity use, 14% to academic travel, and 11% to other activities [13]. William Fox et al. estimated the carbon storage and annual sequestration of campus trees, offering data to support their role in mitigating overall university carbon emissions [14]. He Dongying et al. utilized the emission factor method to estimate carbon emissions for a university in a hot-summer, cold-winter region, discovering that electricity consumption is the principal contributor to campus carbon emissions, accounting for at least 83.43% of the total [15].Stavros et al. compared the carbon dioxide emissions generated by transportation to and from university sports venues during intercollegiate athletic events, and discussed the implications for environmental sustainability and planning [16]. Lorenzo et al. studied the technical and economic feasibility, as well as the carbon emission reduction effects, of installing a photovoltaic distributed generation system at the Polytechnic University of Madrid campus. They proposed a photovoltaic system evaluation method based on data with varying time resolutions, simulation software, and levels of detail [17]. Legorburu and Amanda proposed a new technique that simplifies the lifecycle cost and carbon emission analysis of campus buildings by utilizing observed energy and weather data. The method aims to reduce overall carbon emissions and lifecycle costs by optimizing the selection of the best HVAC system [18]. Alavijeh et al. studied the cost-effectiveness of operational strategies for reducing CO_2_ emissions in local multi-energy systems, using the actual energy system of the Chalmers University Science Park as a case study. They proposed a mixed-integer linear programming multi-objective optimization model, and the results showed that a combined strategy could achieve a 20.8% reduction in emissions with a 2.2% increase in costs [19]. Malaysia et al. evaluated the carbon footprint generated by transportation, electricity, water, and waste production at University Teknologi Malaysia (UTM) over a four-year period, and explored innovative methods for reducing carbon emissions through the utilization of renewable energy [20].

Existing literature predominantly focuses on applying carbon emission factor methods and life cycle assessments to evaluate university carbon footprints, while overlooking the uncertainties inherent in carbon emission inventories. Consequently, current research on carbon emission assessments exhibits limitations in scope coverage.

To address the limitations identified in prior research, this study employs Sichuan University Jinjiang College(hereafter referred to as J University) in Sichuan, China, as a case study and utilizes a hybrid approach combining the emission factor method and the Delphi technique to quantitatively assess carbon emissions in Chinese higher education institutions. This assessment includes direct emissions, indirect emissions, other emissions, and carbon sequestration. Monte Carlo simulation and sensitivity analysis are used to investigate uncertainties in carbon emission inventories and to formulate policy recommendations and strategies for campus carbon reduction. The results of this study provide valuable guidance for universities in developing carbon emission inventories and performing carbon accounting, thereby advancing energy conservation and emission reduction initiatives within higher education institutions.

## 3. Case study: The J university

J University, located in Meishan City, Sichuan Province, China, is a comprehensive higher education institution. The university provides 52 academic programs, with an enrollment of 22,300 students and a faculty of 412 full-time members. Table 1 presents the foundational data for the university for the year 2023.

**Table 1:** Total building area and enrollment data for faculty and students at J University in 2023.

Description	Figure
Total Building Area	380,000 m^2^
Enrolled Students	22,300
Faculty Members	412
Administrative Staff	602
Other Personnel	316
Student-to-Faculty Ratio	22.3:1

The selection of J University for carbon emissions accounting is justified by the following factors:It is situated in southwestern China, within the subtropical humid monsoon climate zone, characterized by hot summers and cold winters, resulting in substantial seasonal fluctuations in energy consumption, including electricity and heating;The university has a high population density and encompasses a range of facilities, including classrooms, dormitories, dining halls, laboratories, and office buildings, as well as extensive campus greenery, which makes it representative of numerous universities across China. According to the “Greenhouse Gas Accounting System” [21] considering existing literature along with the specific characteristics of the campus, emission sources are categorized into direct, indirect, and other types of emissions. Furthermore, to ensure the accuracy of the data, both the carbon sequestration by campus trees and greenery and the uncertainties in the carbon emission inventory were taken into account [22,23].

**Fig 1:**
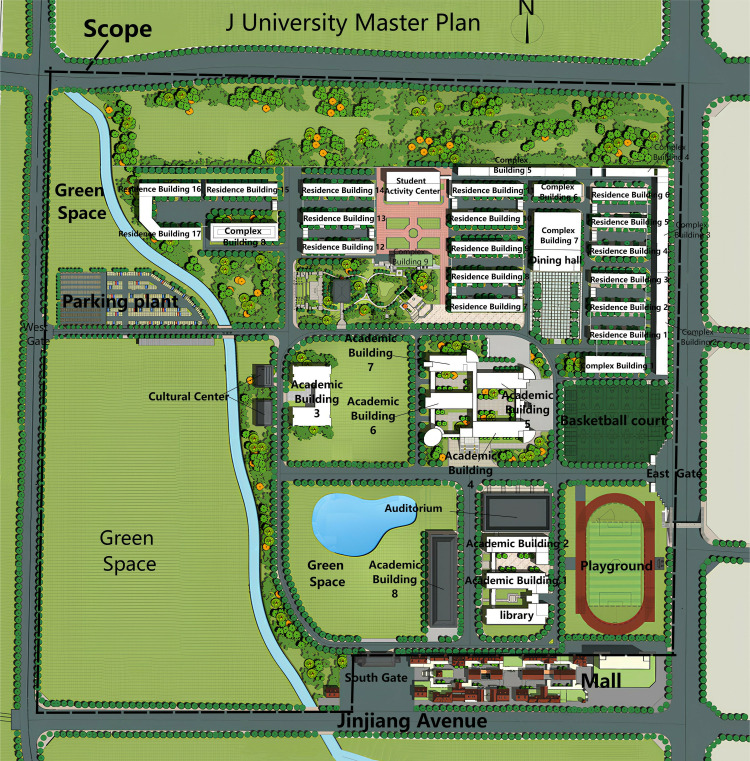
J University total boundary.

This study evaluates the carbon footprint of J University’s campus, encompassing 8 academic buildings, 18 dormitories, 6 multi-purpose buildings, 1 library, 1 dining hall, 1 cultural center, 1 courier service center, and campus greenery (Fig 1).

## 4. Method of calculation

According to the Kyoto Protocol, greenhouse gases are categorized into six types: carbon dioxide (CO_2_), methane (CH_4_), nitrous oxide (N_2_O), hydrofluorocarbons (HFCs), perfluorocarbons (PFCs), and sulfur hexafluoride (SF_6_) [24]. The case study of J University encompasses all six of these gases. For computational convenience, the emissions of the remaining five gases are expressed in terms of carbon dioxide equivalents for statistical analysis.

### 4.1 Carbon emission factor method

The emission factor method is a widely utilized technique for calculating carbon emissions, as outlined in Eq 1 [25]:


$$C=\underset{i}{\sum }C_{i}=\underset{i}{\sum }E_{i}\times X_{i}$$ (1)

In this context, “C” represents the carbon dioxide emissions (measured in ten thousand tons); “i”denotes a specific project; “E_i_”refers to the amount of consumption; and “X_i_” specifies the carbon emission factor.

In this study, the emission factor method is utilized to quantify the carbon emissions from various sources, including electricity, natural gas, commuting and travel, food, heating, water, paper, printing, and school vehicles.Data from 2023, provided by the campus logistics department. Data concerning electricity, natural gas, heating, and water consumption were obtained from instrument readings, whereas additional statistical data were gathered through campus surveys. Furthermore, in January 2024, a survey was administered to all faculty and students to collect data on commuting, travel, and food consumption for the year 2023. A total of 12,300 valid responses were obtained.

Table 2 provides the carbon emission factors corresponding to each of these sources. Notably, the campus’s recyclables, trees, and green spaces contribute to partial carbon offsets [26,27]. The carbon offset factors for each of these sources are presented in Table 3.

**Table 2:** Carbon emission factor of each category.

GHG Emission Source	figure	Unit	Source
Natural Gas	53.06	kg CO₂e/MMBtu	EPA (2024)
Gasoline	8.89	kg·CO₂e/gal	EPA (2024)
Diesel oil	10.21	kg·CO₂e/gal	EPA (2024)
Electricity	0.92	kg·CO₂e/kWh	IPCC (2023)
Heat	0.2~0.6	kg·CO₂e/kWh	Carbon Trust (2024)
Water Supply	0.34	kg·CO₂e/m³	Li, Ruishi et al. [28]
Printing	2.3	kg·CO₂e/kg/Paper	Carbon Trust (2024)
Food Grains	2.5~10	kg·CO₂e/kg	Our World in Data (2023)
Vegetables	2	kg·CO₂e/kg	Our World in Data (2023)
Meat	25	kg·CO₂e/kg	Our World in Data (2023)
Milk	1.3	kg·CO₂e/L	Our World in Data (2023)
Eggs	4.8	kg·CO₂e/kg	Our World in Data (2023)
Fruits	1.1	kg·CO₂e/kg	Our World in Data (2023)
Fertilizer	1.5~3	kg CO₂e/kg	Brentrup, F. et al. [29]
Paper	1.2	kg·CO₂e/kg	Carbon Trust (2024)
Train	0.041	kg·CO₂e/passenger/km	Carbon Trust (2024)
Airplane	0.285	kg·CO₂e/passenger/km	Carbon Trust (2024)
Private Car	0.192	kg·CO₂e/passenger/km	Carbon Trust (2024)
Laboratory chemicals	3~5	kg CO₂e/kg	ISO 14040 (2006)
Public Bus	0.089	kg·CO₂/passenger-kilometer	Carbon Trust (2024)

**Table 3:** Carbon compensation factor of each category.

Substance	Figure	Unit	Source
Glass	−0.31	t·CO₂e·t^−1^	Turner, David A. et al. [30]
Paper	−0.46	t·CO₂e·t^−1^	Turner, David A. et al. [30]
Can	−3.58	t·CO₂e·t^−1^	Turner, David A. et al. [30]
Plastic	−0.24	t·CO₂e·t^−1^	Zheng and Sangwon [31]
Trees	−2~–6	t·CO₂e·ha^−1^·yr^−1^	GEF
Green space	−1~–3	t·CO₂e·ha^−1^·yr^−1^	EPA

### 4.2 The modified Delphi method

The complexity of wastewater treatment processes, combined with regional differences and varying treatment methods, can significantly influence carbon emissions. The direct application of the emission factor method may result in considerable inaccuracies, particularly because wastewater treatment occurs off-campus, making precise data collection challenging. Consequently, this study utilizes the Delphi method to evaluate carbon emission data from regional wastewater treatment plants [32], estimating the carbon emission factor for wastewater treatment based on averaged values.Two rounds of expert consultation with 15 experts in the field of environmental science and carbon accounting (15 experts in the first round and 10 experts in the second round) were conducted to estimate the carbon emission factors of wastewater treatment processes. Data collection and analysis through structured questionnaires to reach consensus on emission factors.

#### 4.2.1 The modified delphi survey questionnaire development.

The modified Delphi method, through multiple rounds of questionnaires and feedback mechanisms, enables the systematic collection of expert opinions [33–35]. In each round, experts can reconsider and adjust their views based on the summary results from the previous round, leading to a gradual convergence and refinement of opinions [36–38]. Furthermore, the anonymity of participants can be maintained, which avoids potential biases or dominant behaviors arising from the exposure of expert identities, ensuring that each expert’s opinion is independently and equally considered. This enhances the objectivity and reliability of the results [39–41]. Most importantly, the modified Delphi method employs various statistical indicators to comprehensively analyze expert opinions, which not only assesses the consistency of the opinions but also delves into the characteristics and patterns of the data, providing robust support for decision-making.

In this study, two rounds of surveys were prepared. The first-round questionnaire focused on three main themes: wastewater treatment process relevance, carbon emission influencing factors, and emission factor estimation. The questionnaire consisted of eight questions, some of which were further detailed into sub-items and open-ended questions (Table 4). After the first round, responses from the participants were collected and anonymized. The second round was based on the results from the first round, providing an initial average emission factor for wastewater treatment, which was then fed back to the experts participating in the second round. If experts had differing opinions, further feedback and adjustments were made until consensus was reached. Table 4 summarizes the questions and references used to design the submitted questionnaire.

**Table 4:** Wastewater Treatment Emission Coefficients Quetionnaire (Improved Delphi Method).

Theme	Question Group	Question Elements & Types	Expert Participation Rounds	References
Participant Basic Information	Personal information of the expert panel (Name (anonymized);Affiliation (related to research); Years of experience; Education; Age; Gender)	First and second rounds: 6 short open-ended questions, mainly to understand the participants’ knowledge of wastewater treatment	1,2	Yun et al. [42]
Wastewater Treatment Processes	1.Among the common wastewater treatment processes (e.g., activated sludge, biofilm, anaerobic treatment), which one is most representative in terms of greenhouse gas emissions?	4 options: Choose one (Activated Sludge, Biofilm, Anaerobic Treatment, Other methods (explain reason))	1	Braden et al. [43]
	2.Are there significant differences in greenhouse gas emissions between small, medium, and large-scale wastewater treatment plants? If yes, how are these differences reflected??	3 options: Choose one (Yes or No, and explain the differences)		
	3. What are the advantages and disadvantages of emerging wastewater treatment processes (e.g., MBR, SBR) compared to traditional processes in terms of greenhouse gas emissions?	Open-ended question, based on participant’s personal experience		
Carbon Emission Influencing Factors	1. What are the main factors influencing carbon emissions in the wastewater treatment process?	6 options: Choose one (If there are other influencing factors, please explain)	1	Friis-Holm and Kristian [44]
	2.Based on the factors mentioned above, which factor has the most significant impact on carbon emissions?	Choose the key factor and briefly explain the reason		
	3. Assess the impact of major pollutants in the influent (e.g., COD, BOD, ammonia nitrogen) on greenhouse gas emissions	Fill in a table with high, medium, or low impact, and explain the reason		
Emission Coefficient Estimation	1. Based on your experience, estimate the greenhouse gas emission coefficient range for conventional urban domestic sewage treatment	5 options: Choose one (A. 0–5, B. 6–10, C. 11–15, D. 15–20, E. above 20)	1	Selene et al. [45]
	2. Based on the chosen range, provide the most likely emission coefficient value	Fill in the value and explain the reason		
Feedback and Adjustment	1. First round expert opinions collection and statistical analysis to obtain preliminary results on greenhouse gas emissions coefficients from wastewater treatment	Feedback results and collect participants’ feedback (Agree, Disagree (explain reasons for disagreement))	2	I-Chieh et al. [46]
	2. Based on the preliminary results, do participants need to adjust the emission coefficient values they previously provided?	2 options: Choose one (Yes (provide adjusted emission coefficient and reason), No)		
Consensus Building	1. What additional factors should be considered or what further research is needed to determine the greenhouse gas emission coefficient for wastewater treatment processes?	Open-ended question	2	Shwu-Feng et al. [47]
	2. What measures can be taken to achieve broader consensus in this field?	Open-ended question		

#### 4.2.2 Recruitment of experts.

Due to the highly specialized nature of the survey, experts outside the relevant research fields would not be able to provide accurate responses. Therefore, the research team employed field surveys and distributed questionnaires to recruit experts for the MDA.

A total of 15 experts in the fields of environmental science and carbon accounting were recruited. All 15 participants took part in the first-round questionnaire, while 10 participants (66.7% of the initial sample) from the first round participated in the second round. Participants filled out the questionnaires based on their own experiences, and for open-ended questions, they provided corresponding responses. Table 5 summarizes the demographic and professional characteristics of the experts who participated in this study. The 15 experts and stakeholders who participated in the first-round questionnaire came from various organizations, with diverse ages, years of work experience, and educational backgrounds.

**Table 5:** Socio-Demographic Characteristics of the Expert Panel.The “%” Representative proportion.

Specific Characteristics	Round 1 (N=15)	Round 2 (N=10)
Gender		
Male	10 (66.7%)	7 (70.0%)
Female	5 (33.3%)	3 (30.0%)
Education		
Bachelor’s	2 (13.3%)	2 (20.0%)
Master’s	7 (46.7%)	3 (30.0%)
Doctorate	6 (40.0%)	5 (50.0%)
Age		
22–32	2 (13.3%)	1 (10.0%)
33–43	3 (20.0%)	1 (10.0%)
44–54	3 (20.0%)	2 (20.0%)
55–65	6 (40.0%)	5 (50.0%)
>65	1 (6.7%)	1 (10.0%)
Work Experience		
≤5 years	1 (6.7%)	1 (10.0%)
5–10 years	3 (20.0%)	1 (10.0%)
11–20 years	7 (46.7%)	5 (50.0%)
>20 years	4 (26.6%)	3 (30.0%)
Workplace		
University	7 (46.7%)	6 (60.0%)
Government (Wastewater Treatment)	3 (20.0%)	2 (20.0%)
NGO (Wastewater Treatment)	3 (20.0%)	1 (10.0%)
Company	2 (13.3%)	1 (10.0%)

#### 4.2.3 Estimation of carbon emission factors for wastewater treatment.

A statistical analysis of the results from the two rounds of surveys (Table 6) reveals that 8 experts (53.3% of the total participants) estimated the emission factor range to be between 10 and 15, 5 experts (33.3%) estimated it to be between 15 and 20, 1 expert (6.67%) chose the range of 5–10, and 1 expert (6.67%) selected the range above 20. Based on the first-round results, the highest most likely value of 20.24 and the lowest value of 9.78 were excluded (with the aim of enhancing data stability by considering the opinions of the majority). After this adjustment, 13 valid results remained, and the calculated average value was 14.77. After incorporating feedback and adjustments, the final value was determined to be 14.64. Therefore, this value is considered more reasonable for estimating the carbon emission factor of wastewater treatment in the region under study.

**Table 6:** Statistical Analysis of Survey Results.

Expert Number	Participation Rounds	Estimated Emission Factor (t·CO₂e/t/d)	Feedback and Adjustments (t·CO₂e/t/d)	Consensus Reached (t·CO₂e/t/d)
1	1,2	Estimated emission factor range: 10–15, Most likely value: 14.58	Agreement with result	14.64
2	1	Estimated emission factor range: 15–20, Most likely value: 15.63	Based on data provided by the workplace	
3	1,2	Estimated emission factor range: 10–15, Most likely value: 13.79	Disagreement with result, adjustment needed, adjusted value: 13.36	The city’s wastewater treatment plant is small-scale with limited capacity, so the value should be lower.
4	1,2	Estimated emission factor range: 10–15, Most likely value: 14.92	Agreement with result	14.64
5	1,2	Estimated emission factor range: 5–10, Most likely value: 9.78	Based on data from the anaerobic treatment process at the wastewater treatment plant	14.64
6	1,2	Estimated emission factor range: 10–15, Most likely value: 12.77	Based on integrated calculations from related research	14.64
7	1,2	Estimated emission factor range: 10–15, Most likely value: 14.20	Based on related research	14.64
8	1	Estimated emission factor range: 15–20, Most likely value: 15.68	Based on data from the activated sludge process at the wastewater treatment plant	
9	1,2	Estimated emission factor range: 10–15, Most likely value: 13.69	Based on data provided by the workplace	14.64
10	1,2	Estimated emission factor range: 15–20, Most likely value: 15.88	Considering anaerobic treatment, where some gases can be “carbon absorbed”, data reflects the value after carbon absorption	14.64
11	1	Estimated emission factor range: above 20, Most likely value: 20.24	Based on data provided by a non-governmental organization at the wastewater treatment plant	
12	1,2	Estimated emission factor range: 10–15, Most likely value: 11.76	Based on related research	14.64
13	1,2	Estimated emission factor range: 15–20, Most likely value: 17.76	Based on related research	14.64
14	1	Estimated emission factor range: 15–20, Most likely value: 16.54	Based on related research	
15	1	Estimated emission factor range: 10–15, Most likely value: 14.79	Based on research from related government departments	

### 4.3 Carbon emission factor estimation for waste treatment

Carbon emissions associated with waste management include transportation, waste processing, leachate treatment, and the electricity consumption of waste processing facilities [48–50]

Carbon emissions resulting from transportation are computed using Eq 2 [51].


$$E_{CO_{2}}=F\times EF$$ (2)

In this equation, “ECO₂” represents the carbon emissions (in kilograms of CO₂), “F” denotes the fuel consumption, and “EF” indicates the fuel emission factor.

Since the waste treatment facility is not located on campus, the carbon emission data for waste processing, leachate treatment, and the electricity consumption of the processing facilities were obtained from the waste treatment station to ensure data accuracy.

The carbon emission factor for waste processing can be estimated using Equation (2) and the data supplied by the waste treatment facility. The relevant data from the waste treatment facility are presented in Table 7.

**Table 7:** Carbon Emission Data for Waste Treatment Facility.

Description	Units	Value
Daily waste processing capacity	Tons per day (t/d)	150
Carbon emissions from incineration and landfilling	Tons CO₂ per ton per day (t·CO₂e/t/d)	1.86
Carbon emissions from leachate treatment	Tons CO₂ per ton per day (t·CO₂e/t/d)	0.11
Carbon emissions from electricity consumption	Tons CO₂ per ton per day (t·CO₂e/t/d)	14.5

### 4.4 Uncertainty analysis

The uncertainty in campus carbon emissions primarily stems from variability in activity data and emission factors [52,53]. This study utilizes the Monte Carlo simulation method, as recommended by the IPCC, and employs the *AUVtoolpro* software to evaluate the uncertainty associated with the total campus carbon emissions.

The assessment of uncertainty using the Monte Carlo method with *AUVtoolpro* involves three main steps: developing the model by specifying variables and parameters; computing emissions for each category based on the inventory data; and executing 10,000 simulations to derive probability distributions for various emission categories or total emissions, along with the relevant statistical data required for uncertainty analysis.

## 5. Results

This section presents the carbon emissions results for each part, the total carbon emissions result, and the uncertainty analysis results.

### 5.1 The carbon emissions from each part

#### 5.1.1. Electricity.

**Fig 2:**
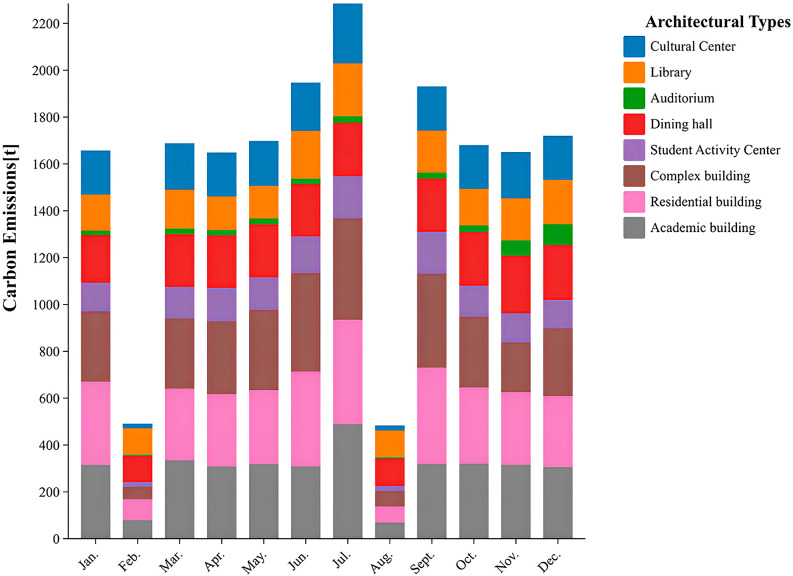
Monthly variations in electricity carbon emissions and the carbon emission intensity of different building types.

The monthly carbon emissions of different types of buildings are due to varying electricity consumption, as shown in Fig 2.The results show that the carbon emissions from buildings were highest in June. Among all types of buildings, the dormitory had the highest carbon emissions intensity from electricity, followed by the teaching building, the general building, the cafeteria, the cultural center, the library, the student activity center, and the auditorium, in descending order.

#### 5.1.2. Natural gas and thermal.

**Fig 3:**
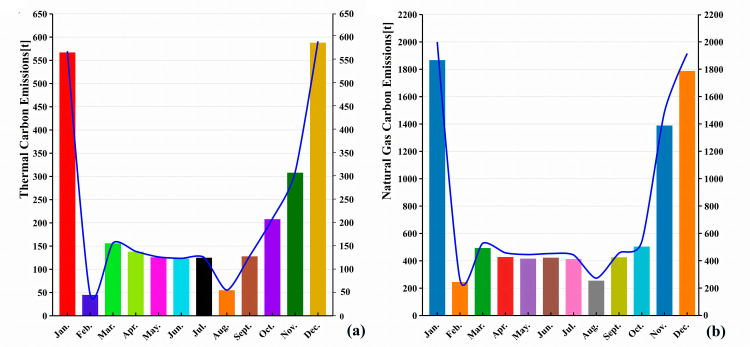
Monthly carbon emissions from thermal energy (a) and natural gas (b).

The monthly carbon emissions from natural gas and heating are shown in Fig 3.The results show that the carbon emissions from natural gas and heating exhibit significant seasonality, with higher emissions in winter and lower emissions in spring, summer, and autumn.

#### 5.1.3. Foods.

**Fig 4:**
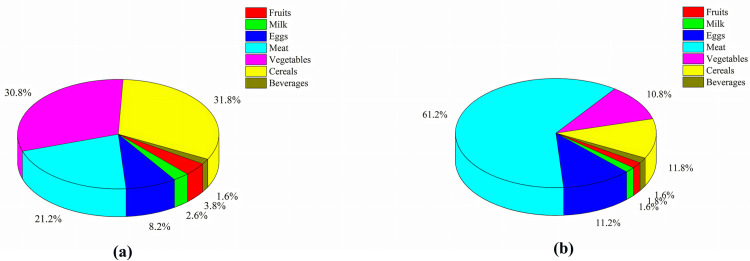
proportion of food consumption (a) and proportion of carbon emission from food consumption (b)in J Universities.

The monthly food consumption ratio of faculty and students on campus and the carbon emissions share generated by the consumption of various foods are shown in Fig 4.The results show that although the consumption of vegetables accounts for the largest proportion, the carbon emissions from meat are the highest.

#### 5.1.4. Wastewater treatment.

**Fig 5:**
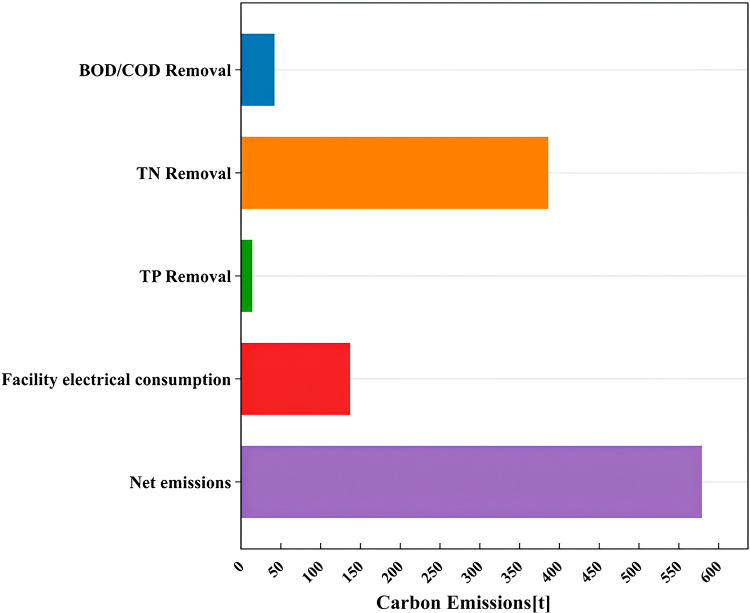
Carbon emissions from wastewater treatment.

The facility employs the AAO (Anaerobic-Anoxic-Oxic) process to address organic matter (BOD/COD), total nitrogen (TN), total phosphorus (TP), suspended solids (SS), ammonia nitrogen (NH₃-N), and other contaminants [39].The carbon emissions are shown in Fig 5.he results show that the main source of carbon emissions in wastewater treatment is the removal of TN.

#### 5.1.5. Automotive commuting and university owned vehicles.

**Fig 6:**
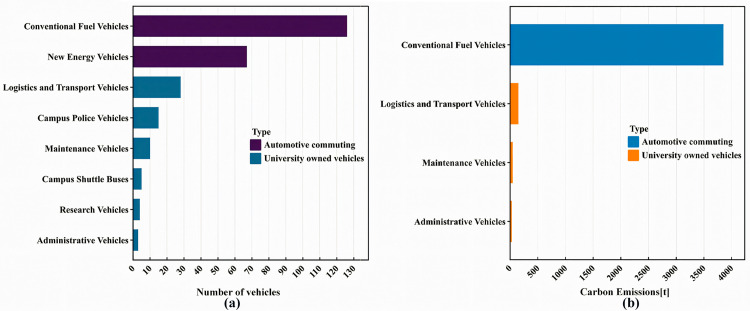
The number of different types of vehicles (a) and Carbon emissions (b).

The number and types of vehicles on campus, along with their carbon emissions, are shown in Fig 6.The results show that the carbon emissions from fossil fuel vehicles are the highest, while the use of new energy vehicles can reduce carbon emissions.

#### 5.1.6. University related travel.

**Fig 7:**
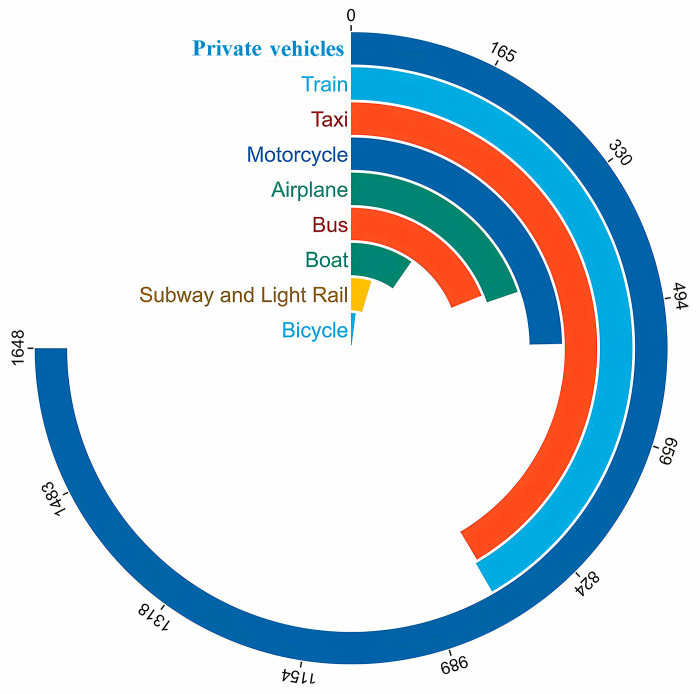
Travel modes and Associated Carbon Emissions. Emissions from air travel, rail travel, shipping, public buses, and subways are influenced by various factors and cannot be precisely quantified, they estimated based on expenses and travel distances [54].

Greenhouse gas emissions related to different modes of transportation for faculty and students (excluding food and accommodation) are shown in Fig 7. The results show that the emissions generated by using personal cars for commuting are the highest.

#### 5.1.7. Waste and recycling transportation.

**Fig 8:**
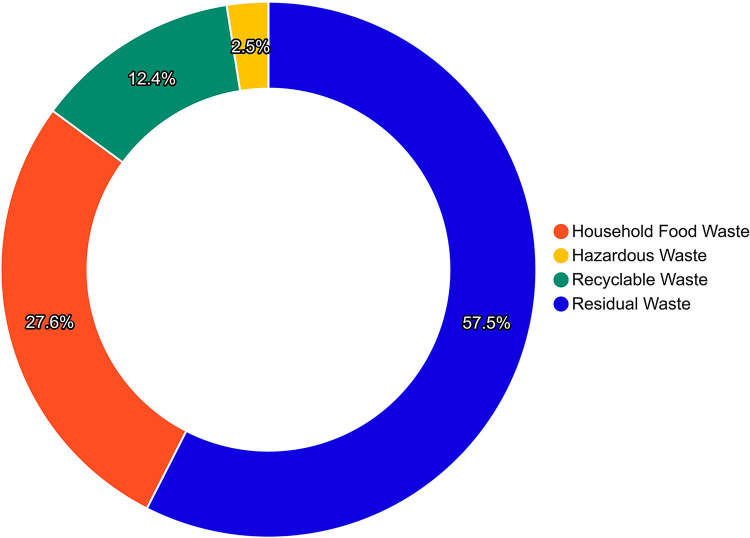
The Composition of University Waste in 2023.

**Fig 9:**
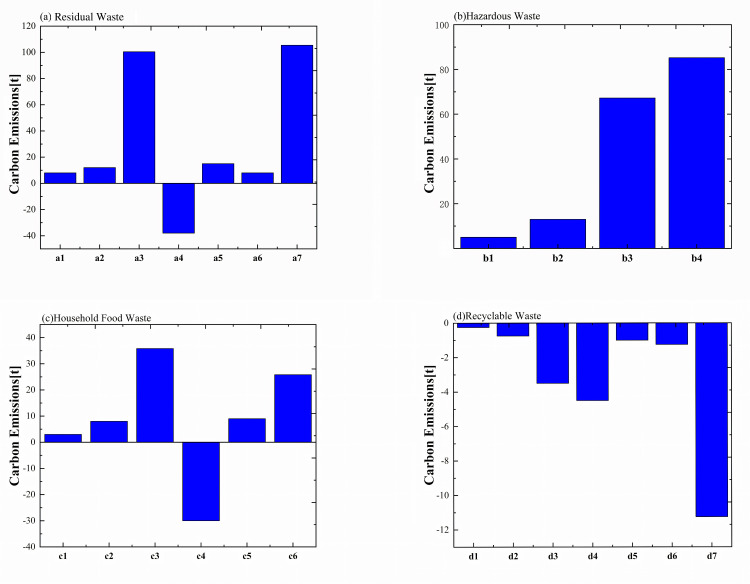
The carbon emissions from waste treatment in 2023 (a1) Transportation, (a2)Facility electricity consumption, (a3) Methane leakage,(a4) Methane powergeneration, (a5) Methane flaring, (a6) Leachate treatment, (a7) Net emissions; (b1)Transportation, (b2) Facility electricity consumption, (b3)Hazardous waste incineration, (b4) Net emissions; (c1) Transportation, (c2) Facility electricity consumption, (c3) Methane leakage, (c4) Methane power generation, (c5) Leachate treatment, (c6) Net emissions; (d1) Transportation, d2.Glass, (d3) Paper, (d4) Aluminum cans, (d5) corrugated cardboard, (d6) Plastics, (d7) Net emissions.

The proportion of different types of waste and the carbon emissions from waste treatment are shown in Figs 8 and 9. The results show that the treatment of other waste accounts for the largest amount, with the highest emissions. The net carbon emissions generated per ton of different types of waste, from highest to lowest, are as follows: other waste (105.42 t)> hazardous waste (85.23 t)> kitchen waste (25.82 t)> recyclables (−11.25 t). The carbon emissions per unit weight of hazardous waste are much higher than those of kitchen waste and other waste.

#### 5.1.8. Paper.

The survey indicates that the primary types of paper used at J University include tissue paper and various forms of copy paper (such as books, exam papers, and other printed materials). Utilizing the carbon emission factor method, which considers the emission factors and carbon offset coefficients specific to these paper types, along with the emission factors associated with printing, it was determined that J University consumed 65.8 tons of paper in 2023 (data provided by the university’s logistics department), resulting in the emission of 124.43 tons of greenhouse gases.

#### 5.1.9. Fertilizer.

In 2023, J University applied a total of 11.11 tons of compound fertilizer (data provided by J University’s logistics department), leading to the emission of 25 tons of greenhouse gases.

#### 5.1.10. Green space and trees.

J University has planted a total of 30.99 hectares of green spaces and trees to enhance the campus environment and reduce greenhouse gas emissions, effectively decreasing emissions by 149.1 tons.

### 5.2 The total carbon emissions result

**Fig 10:**
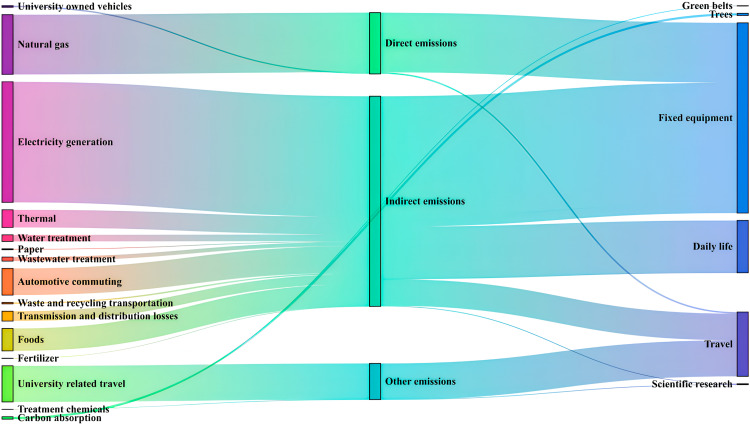
The carbon footprint of J University by source.

In 2023, the total carbon emissions of J University’s campus amounted to 44,733.43 tons of CO₂ equivalent. These emissions comprised direct emissions totaling 8,872.61 tons, indirect emissions of 30,627.22 tons, other emissions amounting to 5,233.6 tons, and carbon sequestration of 149.1 tons, resulting in a net carbon emission of 44,584.33 tons. According to Table 8 and Fig 10, the five largest sources of emissions at J University, ranked from highest to lowest, are electricity (42.34%, including transmission and distribution losses), natural gas (19.40%), travel (11.72%), campus commuting (8.64%), and food (7.73%).

**Table 8:** Greenhouse gas emissions and absorption for J University.

	Source	GHG Emissions (metric tons CO₂-e)
Direct emissions (Scope 1)	University owned vehicles	225.36
	Natural gas	8,647.25
	Sub-total	8,872.61
Indirect emissions (Scope 2)	Electricity generation	17,456.42
	Thermal	2,566.63
	Water treatment	950.45
	Paper	124.43
	Wastewater treatment	578.55
	Automotive commuting	3,852.33
	Waste and recycling transportation	205.22
	Transmission and distribution losses	1,423.52
	Foods	3,444.67
	Fertilizer	25
	Sub-total	30,627.22
Other emissions (Scope 3)	University related travel	5,224.55
	Treatment chemicals	9.05
	Sub-total	5,233.6
Carbon absorption	Green space	−36.88
	Trees	−112.22
	Sub-total	−149.1
	Total	44,584.33

### 5.3 Uncertainty and correlation analysis.

**Fig 11:**
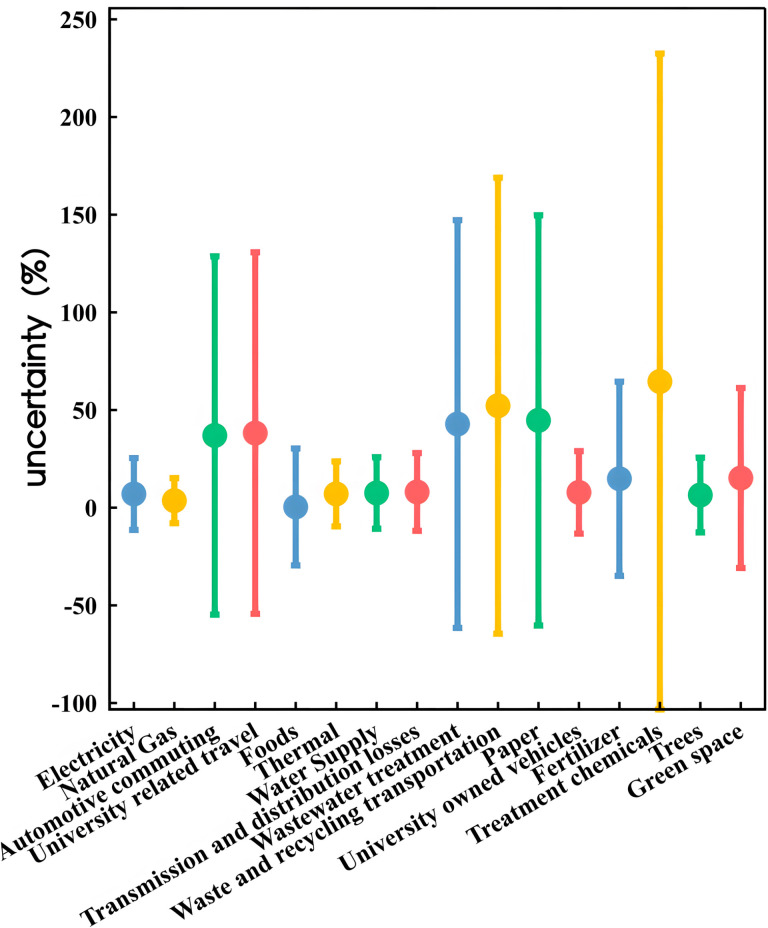
Analysis of uncertainty.

Fig 11 present the results of the Monte Carlo simulations, while Table 9 lists the outcomes of the uncertainty and sensitivity analyses.The analysis reveals that the uncertainty ranges are notably large for business travel, commuting, wastewater treatment, waste management, laboratory chemical handling, and fertilizer use. In contrast, the uncertainty ranges for the remaining carbon emissions inventory are relatively small.

**Table 9:** Emission/absorption source uncertainty range and correlation coefficient.

Name	Uncertainty/ (%)	Correlation
Electricity	−11.37–25.40	0.545
Natural Gas	−7.92–15.15	0.540
Automotive commuting	−54.73–128.67	0.551
University related travel	−54.35–130.73	0.591
Foods	−29.54–30.32	0.543
Thermal	−9.55–23.69	0.521
Water Supply	−10.76–25.83	0.054
Transmission and distribution losses	−11.86–27.95	0.039
Wastewater treatment	−61.55–147.22	0.009
Waste and recycling transportation	−64.51–168.91	0.054
Paper	−60.31–149.69	0.045
University owned vehicles	−13.28–28.94	0.006
Fertilizer	−34.89–64.51	0.003
Treatment chemicals	−103.25–232.46	0.002
Trees	−12.64–25.58	0.076
Green space	−30.88–61.26	0.034

## 6. Discussion

This section will discuss the carbon emissions accounting results and uncertainty analysis, and compare the carbon emissions of universities in different regions.

### 6.1 Discussion of the accounting results

Electricity is the primary source of emissions, with a total emission of 17,456.42 tCO_2_e due to electricity consumption in this study. This electricity is mainly used to support daily operations, teaching, research, as well as campus infrastructure and scientific equipment functions. To reduce electricity consumption, optimization of building materials, structures, and designs can be implemented, such as incorporating energy-efficient designs (e.g., energy-saving designs for new buildings), utilizing renewable energy (e.g., installing photovoltaic panels on campus buildings), and introducing intelligent building management systems (IBMS) [55].

Carbon emissions from natural gas and heating, primarily due to daily dining and winter heating, are the second highest after electricity emissions, at 8,647.25 tCO_2_e and 2,566.63 tCO_2_e, respectively, showing strong seasonal variation. J University can reduce natural gas and heating consumption during the heating season by implementing measures such as centralized heating, zoned heating, installing waste heat recovery systems, and using high-efficiency insulation materials [56,57].

Despite most students at J University residing on campus, commuting by faculty and campus transportation still resulted in 5,449.91 tCO_2_e emissions, primarily due to the use of fossil fuel-powered vehicles. Therefore, promoting the use of new energy vehicles to replace traditional fuel-powered cars can significantly reduce campus carbon emissions [58].

Furthermore, although the campus greening coverage is 45%, the carbon absorption is only 149.1 tCO_2_e, accounting for just 0.33% of the total emissions. Therefore, relying solely on campus greening to achieve carbon neutrality is unrealistic [59].

### 6.2 Uncertainty and correlation analysis

The high uncertainty in business travel and commuting is primarily due to the use of survey-based data collection methods, which inherently introduce some degree of uncertainty [60]. For wastewater treatment, the uncertainty mainly arises from the variability in emission factors [61]; the carbon emission factor for wastewater treatment was estimated using data provided by wastewater treatment plants and the Delphi method. Additionally, as waste management is conducted off-campus, the carbon emission factor for waste management was also estimated based on data provided by waste management facilities. Consequently, the uncertainties for wastewater and waste management are substantial. The handling of laboratory chemicals involves a wide variety of substances, requiring complex and categorized disposal processes, and the use of emission factors from existing studies that may not perfectly align with actual conditions contributes to significant uncertainty [62,63]. Similarly, for fertilizers, the use of emission factors from existing research, which may differ from actual emission factors, results in considerable uncertainty.

It is noteworthy that data for electricity, natural gas, and water consumption come from instrument readings such as meters, leading to smaller uncertainty ranges [20]. This indicates that the method of obtaining activity data and the selection of emission factors have a significant impact on the uncertainty of the results. To minimize errors, it is crucial to choose appropriate methods for obtaining activity data and emission factors.

Regarding correlation coefficients, a higher value indicates a greater contribution of that emission source to the total uncertainty in carbon emissions [64,65]. The results show that commuting and business travel have the highest correlation coefficients due to their significant carbon emissions and high uncertainty. This is followed by electricity, which, despite its lower uncertainty, has a carbon emission volume much higher than other sources. The uncertainty in natural gas and food is similar to electricity due to comparable factors. Other emission sources, having relatively small carbon emissions, exhibit lower correlation coefficients. To enhance the accuracy and reliability of carbon emissions calculations, it is essential to focus on sources with high carbon emissions to control the overall uncertainty in carbon emissions.

### 6.3 Comparison of carbon emissions among universities in different regions

To explore the differences in carbon emissions among universities, this study conducts a comparative analysis of campus carbon emissions globally, as shown in Table 10. In terms of both total and per capita carbon emissions, J University ranks slightly above average. This discrepancy arises from variations in the scope of carbon accounting across institutions. For instance, Stanford University has undertaken a comprehensive assessment of campus greenhouse gas emissions in pursuit of sustainability, implementing reduction measures for direct and indirect emissions since 2021. However, strategies for mitigating other emissions—such as those from business travel, student travel, and employee commuting—have been less clearly defined due to the complexity and broad scope of data collection and management. Nonetheless, Stanford University developed specific strategies for these other emissions in 2022, aiming for net-zero emissions across all scopes by 2050. The extensive campus area, high-tech equipment, laboratories, and frequent academic travel contribute to Stanford University’s notably higher per capita carbon emissions compared to some other universities [66]. Conversely, the National University of Colombia’s carbon accounting focuses primarily on transportation, wastewater treatment, electricity consumption, and email emissions, resulting in significantly lower per capita carbon emissions due to the narrower scope of its accounting [67]. Thus, developing a standardized carbon footprint accounting method applicable to universities worldwide could enhance comparability of campus carbon emissions across different regions and support the formulation of effective carbon reduction plans [68].

**Table 10:** University carbon emission comparison.

University Name	Country	Emission Levels(t CO₂e)	Per Capita Emissions (t/person)	Year	Source
University of Cambridge	UK	102,049.9	3.5	2016	Cano et al. [67]
University of Madrid School of Forestry Engineering	Spain	2147	1.87	2010	Cano et al. [67]
University of California, Berkeley	Mexico	151,650	2.9	2016	Cano et al. [67]
Stanford University	USA	102,230	3.41	2021	Stanford University [72]
University of Alberta	Canada	32,5351	6.51	2012	Cano et al. [67]
Bournemouth University	UK	29892	1.41	2019	Vasiliki et al. [73]
University of Oulu	Finland	19,072	1.13	2019	André et al. [74]
National University of Colombia	Colombia	7251	0.432	2019	Cano et al. [67]
Clemson University	USA	95,000	4.4	2014	Raeanne et al. [75]
Beijing Forestry University	China	43,249	1.52	2021	Cao and Zhang [76]
J University	China	44584	1.89	2023	This Study

The climate zone of a university also impacts its carbon emissions. For example, both J University and Beijing Forestry University experience distinct seasons. However, J University does not provide centralized heating during winter, whereas Beijing Forestry University does, resulting in significantly lower heating-related carbon emissions at J University. Conversely, during the summer, the extended high temperatures in Sichuan lead to higher cooling-related carbon emissions at J University compared to Beijing Forestry University. Additionally, the National University of Colombia, located near the equator with an average annual temperature of 14°C [69], does not require heating in winter, thus substantially reducing heating-related carbon emissions.

A comparison of per capita carbon emissions among universities worldwide reveals that those in developed countries exhibit significantly higher per capita emissions than their counterparts in other regions. This difference is primarily due to advanced research equipment and the prevalence of off-campus housing in developed countries [70,71]. For example, Stanford University’s 2021 Environmental and Sustainability Annual Report highlights that electricity consumption by research equipment remains a major focus for reduction. Although strategies for direct and indirect emissions have been established, strategies for other emissions (mainly from commuting and travel) are challenging to develop due to the use of off-campus housing, which generates substantial emissions from commuting and travel.

## 7. Conclusion

In the context of advancing global sustainable development goals, universities are encouraged to actively engage in carbon emissions accounting and analysis. This study utilized a combination of the emission factor method and the Delphi method to estimate the net carbon emissions of J University in China for the year 2023. To assess the uncertainty and sensitivity of campus carbon emissions, the Monte Carlo simulation method, as recommended by the IPCC, was applied. The results show that J University’s net carbon emissions totaled 44,584.33 tons of CO₂ equivalent (tCO₂e), with per capita emissions amounting to 1.89 tCO₂e. The primary sources of carbon emissions on campus, in descending order, were electricity (18,879.94 tCO₂e), natural gas (8,647.25 tCO₂e), business travel (5,224.55 tCO₂e), campus commuting (3,852.33 tCO₂e), food consumption (3,444.67 tCO₂e), and heating (2,566.63 tCO₂e).

This study provides a comprehensive framework for the Chinese universities carbon emissions accounting in academic settings and presents a detailed case study for researchers in this field. It also offers an in-depth exploration of statistical uncertainties, thereby providing a solid scientific basis for accurately calculating carbon emissions in future studies.Although the scope of the study is limited to the campus, it is important to continue research along this line, providing a research foundation for related fields in the future (such as the construction industry), and creating a comparative knowledge system.

## Supporting information

S1 File Questionnaire on greenhouse gas emission factors in wastewater treatment process. (DOCX)

## References

[1] Valls-Val K, Bovea MD. Carbon footprint assessment tool for universities: CO2UNV. Sustain Prod Consum. 2022;29:791–804. doi: 10.1016/j.spc.2021.11.020

[2] Gillingham K, Stock JH. The cost of reducing greenhouse gas emissions. J. Econ. Perspect. 2018;32(4):53–72. doi: 10.1257/jep.32.4.53

[3] Romero JP, Gramkow C. Economic complexity and greenhouse gas emissions. World Dev. 2021;139:105317. doi: 10.1016/j.worlddev.2020.105317

[4] Valls-Val K, Bovea MD. Carbon footprint in higher education institutions: A literature review and prospects for future research. Clean Technol Environ Policy. 2021;23(9):2523–42. doi: 10.1007/s10098-021-02180-2 34456663 PMC8382111

[5] Pan T, Zuo L, Zhang Z, Zhao X, Sun F, Zhu Z, et al. Impact of land use change on water conservation: A case study of zhangjiakou in yongding river. Sustainability. 2020;13(1):22. doi: 10.3390/su13010022

[6] World Meteorological Organization. The State of Greenhouse Gases in the Atmosphere Based on Global Observations through 2022. WMO Greenhouse Gas Bulletin, No.19. 2023.

[7] Wang J, Huang S. Net zero—Carbon transition and multiple synergies—Carbon footprint accounting and carbon neutral pathways optimization in universities. J Hebei Univ Econ Bus, 45(1);2024.

[8] The National Bureau of Statistics of China. China statistical yearbook. 2023

[9] Yang Y, Gao F. Research review on the low-carbon path of built environment in university campus. South Archit. 2024;2(2):53–63.

[10] Sun H. Research on carbon emission control strategy of existing campus of colleges and universities in China. Energy Conserv. 2024;No.05:92–4.

[11] Samara F, Ibrahim S, Yousuf ME, Armour R. Carbon footprint at a united arab emirates university: Ghg protocol. Sustainability. 2022;14(5):2522. doi: 10.3390/su14052522

[12] Ridhosari B, Rahman A. Carbon footprint assessment at Universitas Pertamina from the scope of electricity, transportation, and waste generation: Toward a green campus and promotion of environmental sustainability. J Clean Prod. 2020;246:119172. doi: 10.1016/j.jclepro.2019.119172

[13] Mendoza-Flores R, Quintero-Ramírez R, Ortiz I. The carbon footprint of a public university campus in Mexico City. Carbon Manag. 2019;10(5):501–11. doi: 10.1080/17583004.2019.1642042

[14] Fox W, et al. Estimating carbon stock of live trees located on the main campus of the University of Georgia. J For. 2020;118(5):457–65.

[15] He D. Campus carbon accounting based on emission coefficient method. J. Xi'an Polytech Univ. 2022;36(04):78–83.

[16] Triantafyllidis S, Ries R, Kaplanidou K. Carbon dioxide emissions of spectators’ transportation in collegiate sporting events: Comparing on-campus and off-campus stadium locations. Sustainability. 2018;10(1):241. doi: 10.3390/su10010241

[17] Olivieri L, Caamaño-Martín E, Sassenou L-N, Olivieri F. Contribution of photovoltaic distributed generation to the transition towards an emission-free supply to university campus: Technical, economic feasibility and carbon emission reduction at the Universidad Politécnica de Madrid. Renew Energy. 2020;162:1703–14. doi: 10.1016/j.renene.2020.09.120

[18] Legorburu G, Smith AD. Incorporating observed data into early design energy models for life cycle cost and carbon emissions analysis of campus buildings. Energy Build. 2020;224:110279. doi: 10.1016/j.enbuild.2020.110279

[19] Mirzaei Alavijeh N, Steen D, Norwood Z, Anh Tuan L, Agathokleous C. Cost-Effectiveness of carbon emission abatement strategies for a local multi-energy system—A case study of chalmers university of technology campus. Energies. 2020;13(7):1626. doi: 10.3390/en13071626

[20] Naderipour A, Abdul-Malek Z, Arshad RN, Kamyab H, Chelliapan S, Ashokkumar V, et al. Assessment of carbon footprint from transportation, electricity, water, and waste generation: towards utilisation of renewable energy sources. Clean Techn Environ Policy. 2021;23(1):183–201. doi: 10.1007/s10098-020-02017-4

[21] Greenhouse Gas Protocol. Corporate Accounting and Reporting Standard (Revised Edition). World Resources Institute and World Business Council for Sustainable Development. 2015. https://ghgprotocol.org/corporate-standard

[22] Setyowati DL. Energy Consumption, Emission Absorption and Carbon Emission Reduction on Semarang State University Campus. Int J Energy Environ Eng. 2019;13(7):505–9.

[23] Hickmann T. Voluntary global business initiatives and the international climate negotiations: A case study of the Greenhouse Gas Protocol. J Clean Prod. 2017;169:94–104. doi: 10.1016/j.jclepro.2017.06.183

[24] Han P, Zeng N, Oda T, Lin X, Crippa M, Guan D, et al. Evaluating China’s fossil-fuel CO₂ emissions from a comprehensive dataset of nine inventories. Atmos Chem Phys. 2020;20(19):11371–85. doi: 10.5194/acp-20-11371-2020

[25] Quan C, Cheng X, Yu S, Ye X. Analysis on the influencing factors of carbon emission in China’s logistics industry based on LMDI method. Sci Total Environ. 2020;734:138473. doi: 10.1016/j.scitotenv.2020.138473 32460061

[26] Jo H-K, Kim J-Y, Park H-M. Carbon reduction and planning strategies for urban parks in Seoul. Urban For Urban Green. 2019;41:48–54. doi: 10.1016/j.ufug.2019.03.009

[27] Magazzino C, Mele M, Schneider N. The relationship between municipal solid waste and greenhouse gas emissions: Evidence from Switzerland. Waste Manag. 2020;113:508–20. doi: 10.1016/j.wasman.2020.05.033 32546447

[28] Li R, et al. Water-energy-carbon nexus at campus scale: Case of North China University of water resources and electric power. Energy Policy. 2022;166. doi: 10.1016/j.jclepro.2017.06.183

[29] Brentrup F, Küsters J, Kuhlmann H, Lammel J. Environmental impact assessment of agricultural production systems using the life cycle assessment methodology. Eur J Agron. 2004;20(3):247–64. doi: 10.1016/s1161-0301(03)00024-8

[30] Turner DA, Williams ID, Kemp S. Greenhouse gas emission factors for recycling of source-segregated waste materials. Resour Conserv Recycl. 2015;105:186–97. doi: 10.1016/j.resconrec.2015.10.026

[31] Zheng J, Suh S. Strategies to reduce the global carbon footprint of plastics. Nat Clim Chang. 2019;9(5):374–8. doi: 10.1038/s41558-019-0459-z

[32] Lopes HS, Remoaldo PC, Ribeiro V, Martín-Vide J. Pathways for adapting tourism to climate change in an urban destination - Evidences based on thermal conditions for the Porto Metropolitan Area (Portugal). J Environ Manage. 2022;315:115161. doi: 10.1016/j.jenvman.2022.115161 35526395

[33] Keegan RJ, Barnett LM, Dudley DA, Telford RD, Lubans DR, Bryant AS, et al. Defining physical literacy for application in australia: A modified delphi method. J Teach Phys Educ. 2019;38(2):105–18. doi: 10.1123/jtpe.2018-0264

[34] Gunduz M, Elsherbeny HA. Operational framework for managing construction-contract administration practitioners’ perspective through modified delphi method. J Constr Eng Manage. 2020;146(3). doi: 10.1061/(asce)co.1943-7862.0001768

[35] Ahmad OF, Mori Y, Misawa M, Kudo S-E, Anderson JT, Bernal J, et al. Establishing key research questions for the implementation of artificial intelligence in colonoscopy: A modified Delphi method. Endoscopy. 2021;53(9):893–901. doi: 10.1055/a-1306-7590 33167043 PMC8390295

[36] Viljoen CA, Millar RS, Manning K, Burch VC. Determining electrocardiography training priorities for medical students using a modified Delphi method. BMC Med Educ. 2020;20(1):431. doi: 10.1186/s12909-020-02354-4 33198726 PMC7670661

[37] Ko W-H, Lu M-Y. Evaluation of the professional competence of kitchen staff to avoid food waste using the modified delphi method. Sustainability. 2020;12(19):8078. doi: 10.3390/su12198078

[38] Bessa A, Maclennan S, Enting D, Bryan R, Josephs D, Hughes S, et al. Consensus in bladder cancer research priorities between patients and healthcare professionals using a four-stage modified delphi method. Eur Urol. 2019;76(2):258–9. doi: 10.1016/j.eururo.2019.01.031 30712969

[39] Wang Y, Wang T, Wang A, Chen S, Jiao L, Shi J, et al. Identifying the competencies of China’s paediatric residents: A modified Delphi method study. BMJ Open. 2021;11(2):e041741. doi: 10.1136/bmjopen-2020-041741 33597133 PMC7893650

[40] Nayahangan LJ, Stefanidis D, Kern DE, Konge L. How to identify and prioritize procedures suitable for simulation-based training: Experiences from general needs assessments using a modified Delphi method and a needs assessment formula. Med Teach. 2018;40(7):676–83. doi: 10.1080/0142159X.2018.1472756 29938547

[41] Rioja-Lang F, Bacon H, Connor M, Dwyer CM. Rabbit welfare: Determining priority welfare issues for pet rabbits using a modified Delphi method. Vet Rec Open. 2019;6(1):e000363. doi: 10.1136/vetreco-2019-000363 31903189 PMC6924855

[42] Wei Y, Wang F, Pan Z, Wang M, Jin G, Liu Y, et al. Development of a competency model for general practitioners after standardized residency training in China by a modified Delphi method. BMC Fam Pract. 2021;22(1):171. doi: 10.1186/s12875-021-01508-7 34433420 PMC8390270

[43] O’Neill B, Aversa V, Rouleau K, Lazare K, Sullivan F, Persaud N. Identifying top 10 primary care research priorities from international stakeholders using a modified Delphi method. PLoS One. 2018;13(10):e0206096. doi: 10.1371/journal.pone.0206096 30359391 PMC6201922

[44] Egfjord KF-H, Sund KJ. A modified Delphi method to elicit and compare perceptions of industry trends. MethodsX. 2020;7:101081. doi: 10.1016/j.mex.2020.101081 33083242 PMC7554649

[45] Valerino-Perea S, Armstrong MEG, Papadaki A. Development of an index to assess adherence to the traditional Mexican diet using a modified Delphi method. Public Health Nutr. 2021;24(14):4387–96. doi: 10.1017/S1368980020004565 33183382 PMC10195309

[46] Hsu I-C, Shih Y-J, Pai F-Y. Applying the modified delphi method and DANP to determine the critical selection criteria for local middle and top management in multinational enterprises. Mathematics. 2020;8(9):1396. doi: 10.3390/math8091396

[47] Tsay S-F, Zedreck Gonzalez JF, Tsay S-L, Tung H-H, Engberg SJ, Hu SH. Develop and validate family nurse practitioner transition program in taiwan by using modified delphi method. Nurse Educ Today. 2021;98:104765. doi: 10.1016/j.nedt.2021.104765 33517183

[48] Sarigiannis DA, Handakas EJ, Karakitsios SP, Gotti A. Life cycle assessment of municipal waste management options. Environ Res. 2021;193:110307. doi: 10.1016/j.envres.2020.110307 33065069

[49] Ahmed N, Matsushima K, Nemoto K, Kondo F. Identification of inheritance and genetic loci responsible for wrinkled fruit surface phenotype in chili pepper (Capsicum annuum) by quantitative trait locus analysis. Mol Breed. 2024;45(1):5. doi: 10.1007/s11032-024-01528-y 39734933 PMC11671457

[50] Wang D, He J, Tang Y-T, Higgitt D, Robinson D. Life cycle assessment of municipal solid waste management in nottingham, england: Past and future perspectives. J Clean Prod. 2020;251:119636. doi: 10.1016/j.jclepro.2019.119636

[51] Liu T, Cao J, Miatto A. Impacts of a municipal solid waste classification policy on carbon emissions: Case study of Beijing, China. J Mater Cycles Waste Manag. 2024;26(4):2478–90. doi: 10.1007/s10163-024-01985-9

[52] Wang S, et al. Life-cycle assessment of carbon footprint of bike-share and bus systems in campus transit. Sustainability. 2022;14(9):4706. doi: 10.3390/su14094706

[53] Song J, Liu Y, Zhuang M, Gu W, Cui Z, Pang M, et al. Estimating crop carbon footprint and associated uncertainty at prefecture-level city scale in China. Resour Conserv Recycl. 2023;199:107263. doi: 10.1016/j.resconrec.2023.107263

[54] Fan J, Meng X, Tian J, Xing C, Wang C, Wood J. A review of transportation carbon emissions research using bibliometric analyses. J Traffic Transp Eng (Engl. Ed.). 2023;10(5):878–99. doi: 10.1016/j.jtte.2023.09.002

[55] Guo Z, Wang Q, Zhao N, Dai R. Carbon emissions from buildings based on a life cycle analysis: Carbon reduction measures and effects of green building standards in China. Low-carbon Mater Green Constr. 2023;1(1). doi: 10.1007/s44242-022-00008-w

[56] Lin XJ, Zhang N, Mao YH, Chen JY, Tian XT, Zhong W. A review of the transformation from urban centralized heating system to integrated energy system in smart city. Appl Therm Eng. 2024;240:122272. doi: 10.1016/j.applthermaleng.2023.122272

[57] Romanchenko D, Nyholm E, Odenberger M, Johnsson F. Impacts of demand response from buildings and centralized thermal energy storage on district heating systems. Sustain Cities Soc. 2021;64:102510. doi: 10.1016/j.scs.2020.102510

[58] Isik M, Dodder R, Kaplan PO. Transportation emissions scenarios for New York City under different carbon intensities of electricity and electric vehicle adoption rates. Nat Energy. 2021;6:92–104. doi: 10.1038/s41560-020-00740-2 34804594 PMC8597912

[59] Tadesse KA, Lu Z, Shen Z, Daba NA, Li J, Alam MA, et al. Impacts of long-term chemical nitrogen fertilization on soil quality, crop yield, and greenhouse gas emissions: With insights into post-lime application responses. Sci Total Environ. 2024;944:173827. doi: 10.1016/j.scitotenv.2024.173827 38866164

[60] Yin F, Chen Y. The robust optimization for p-hub median problem under carbon emissions uncertainty. Conf Proc.

[61] Obotey Ezugbe E, Rathilal S. Membrane technologies in wastewater treatment: A review. Membranes (Basel). 2020;10(5):89. doi: 10.3390/membranes10050089 32365810 PMC7281250

[62] Lan J-F, Yu E. Chemical waste recycle and disposal associated with chemical laboratory teaching in colleges and universities. Univ Chem. 2016;31(8):71–5. doi: 10.3866/pku.dxhx201510028

[63] Li Z, Wang W, Liu Y, Han M, Wei L, Jiao H. Research on the safety management and disposal of chemical laboratory waste. Daxue Huaxue. 2024;0(0):2312090. doi: 10.3866/pku.dxhx202312090

[64] Mattila T, Kujanpää M, Dahlbo H, Soukka R, Myllymaa T. Uncertainty and sensitivity in the carbon footprint of shopping bags. J Ind Ecol. 2011;15(2):217–27. doi: 10.1111/j.1530-9290.2010.00326.x

[65] Muthu SS, Li Y, Hu JY, Mok PY. Carbon footprint of shopping (grocery) bags in China, Hong Kong and India. Atmos Environ. 2011;45(2):469–75. doi: 10.1016/j.atmosenv.2010.09.054

[66] Stanford University. *Sustainability at Stanford: 2022-2023 Year in Review*. Stanford University, 2023.

[67] Cano N, Berrio L, Carvajal E, Arango S. Assessing the carbon footprint of a Colombian University Campus using the UNE-ISO 14064-1 and WRI/WBCSD GHG Protocol Corporate Standard. Environ Sci Pollut Res Int. 2023;30(2):3980–96. doi: 10.1007/s11356-022-22119-4 35962170 PMC9374572

[68] Gu Y, Wang H, Xu J, Wang Y, Wang X, Robinson ZP, et al. Quantification of interlinked environmental footprints on a sustainable university campus: A nexus analysis perspective. Appl Energy. 2019;246:65–76. doi: 10.1016/j.apenergy.2019.04.015

[69] Ghaem Sigarchian S. Design Optimization of a Small-Scale Polygeneration Energy System in Different Climate Zones in Iran. Energies. 2018;11(5):1115. doi: 10.3390/en11051115

[70] Schleich J, Dütschke E, Kanberger E, Ziegler A. On the relationship between individual carbon literacy and carbon footprint components. Ecol Econ. 2024;218:108100. doi: 10.1016/j.ecolecon.2023.108100

[71] Palm J, Darby SJ. The meanings of practices for energy consumption – A comparison of homes and workplaces. Sci Technol Stud. 2014;27(2):72–92. doi: 10.23987/sts.55325

[72] Stanford University. *Sustainability at Stanford: 2021-2022 Year in Review*. Stanford University, 2022.

[73] Kourgiozou V, Commin A, Dowson M, Rovas D, Mumovic D. Scalable pathways to net zero carbon in the UK higher education sector: A systematic review of smart energy systems in university campuses. Renew Sustain Energy Rev. 2021;147:111234. doi: 10.1016/j.rser.2021.111234

[74] Stephan A, Muñoz S, Healey G, Alcorn J. Analysing material and embodied environmental flows of an Australian university — Towards a more circular economy. Resour Conserv Recycl. 2020;155:104632. doi: 10.1016/j.resconrec.2019.104632

[75] Clabeaux R, Carbajales-Dale M, Ladner D, Walker T. Assessing the carbon footprint of a university campus using a life cycle assessment approach. J Clean Prod. 2020;273:122600. doi: 10.1016/j.jclepro.2020.122600

[76] Cao R, Li F, Zhang L. Accounting and analysis of carbon emissions in higher education institutions: A case study of Beijing A University. Environ Sci. 2023. doi: 10.13227/j.hjkx.202305013

