# Supplementary material for: Carbon emissions accounting and uncertainty analysis in campus settings: A case study of a university in Sichuan, China
Source: PLoS One. 2025 Apr 16;20(4):e0321216. doi: 10.1371/journal.pone.0321216 (PMC12002547; doi:10.1371/journal.pone.0321216)
Supplement: S1 File — (DOCX) [file pone.0321216.s001.docx]

**Wastewater Treatment Greenhouse Gas Emission Factor**

**Questionnaire**

Dear Expert,

Thank you very much for taking the time to participate in this research on greenhouse gas emission factors in wastewater treatment processes. The aim of this study is to gather expert opinions through a scientific survey to collaboratively determine a scientifically reasonable emission factor. Your professional insights are essential to our research, and we kindly ask you to complete the following questionnaire based on your experience and expertise.

### I. Basic Information

## 1.Your Name: (Anonymous)

## 2.Work Affiliation:

## A. University

## B. Wastewater Treatment Government Department

## C. Wastewater Treatment Non-Governmental Organization

## D. Enterprise

## 3.Years of Experience:

## A. ≤ 5 years

## B. 5 - 10 years

## C. 11 - 20 years

## D. Over 20 years

## 4.Highest Level of Education:

## A. Bachelor's

## B. Master's

## C. Doctorate

## 5.Age:

## A. 22 - 32

## B. 33 - 43

## C. 44 - 54

## D. 55 - 65

## E. Over 65

## 6.Gender:

## A. Male

## B. Female

### II. Round 1 Questionnaire

### (A) Wastewater Treatment Processes

1.In your opinion, which of the commonly used wastewater treatment processes (e.g., activated sludge, biofilm, anaerobic treatment, etc.) is most representative in terms of greenhouse gas emissions? Please briefly explain your reasoning.
A. Activated Sludge
B. Biofilm
C. Anaerobic Treatment
D. Other (please specify)
Reason:

2.Do significant differences exist in greenhouse gas emissions among wastewater treatment plants of different sizes (small, medium, large)? If so, in which areas are these differences most prominent?
A. Yes
B. No
Differences:

### 3.For emerging wastewater treatment processes such as MBR (Membrane Bioreactor) and SBR (Sequencing Batch Reactor), what are their advantages and disadvantages in terms of greenhouse gas emissions compared to traditional methods? Please provide examples.

### Advantages:

### Disadvantages:

### (B) Carbon Emission Influencing Factors

1.What do you consider to be the main factors affecting carbon emissions in the wastewater treatment process? (Select multiple options if applicable)
A. Treatment Process
B. Influent Quality
C. Energy Consumption
D. Equipment Efficiency
E. Sludge Treatment Methods
F. Other (please specify)

2.Of the factors listed above, which do you think has the most significant impact on carbon emissions? Please briefly explain your reasoning.
Key Factor:

Reason:

3.Please assess the impact of major pollutants in the influent (such as COD, BOD, ammonia nitrogen, etc.) on greenhouse gas emissions and briefly explain your reasoning.

| Pollutant | Impact Level (High, Medium, Low) | Reason |
| --- | --- | --- |
| COD |  |  |
| BOD |  |  |
| Ammonia |  |  |

1. **Emission Factor Estimation**
2. Based on your experience, what is the approximate range of the greenhouse gas emission factor (in CO₂ equivalents, units: t·CO₂e/t/d wastewater) for a conventional urban domestic wastewater treatment plant (e.g., Pengshan)?
   A. 0 - 5
   B. 5 - 10
   C. 10 - 15
   D. 15 - 20
   E. Above 20

2.If you have selected a range, please provide the most likely emission factor value and explain your reasoning.
Most Likely Value:
Reasoning:

# **Wastewater Treatment Greenhouse Gas Emission Factor Questionnaire**

# Dear Expert,

# Thank you very much for taking the time to participate in this research on greenhouse gas emission factors in wastewater treatment processes. This study aims to gather expert opinions through a scientific survey to collaboratively determine a scientifically reasonable emission factor. Your professional insights are crucial to our research, and we kindly ask you to complete the following questionnaire based on your experience and expertise.

# **I. Basic Information**

# 1.Your Name: (Anonymous)

# 2.Work Affiliation:

# A. University

# B. Wastewater Treatment Government Department

# C. Wastewater Treatment Non-Governmental Organization

# D. Enterprise

# 3.Years of Experience:

# A. ≤ 5 years

# B. 5 - 10 years

# C. 11 - 20 years

# D. Over 20 years

# 4.Highest Level of Education:

# A. Bachelor's

# B. Master's

# C. Doctorate

# 5.Age:

# A. 22 - 32

# B. 33 - 43

# C. 44 - 54

# D. 55 - 65

# E. Over 65

# 6.Gender:

# A. Male

# B. Female

# **II. Round 2 Questionnaire**

# **(A) Feedback and Adjustments**

# After collecting and analyzing expert opinions in the first round, we have obtained preliminary results regarding the greenhouse gas emission factor for wastewater treatment processes. The results are provided to you for review (see attachment).

# 1.Do you agree with the preliminary results?

# A. Yes

# B. No

# If you disagree, please indicate the areas where you find issues and explain your perspective.

# 2.Based on the preliminary results and your latest considerations, do you feel the need to adjust the emission factor estimates you previously provided?

# A. Yes

# B. No

# If yes, please provide the adjusted emission factor and the reasoning behind the adjustment.

# Adjusted Value:

# Reasoning:

# **(B) Reaching Consensus**

# 1.In your opinion, what additional factors or studies should be considered in determining the greenhouse gas emission factor for wastewater treatment processes?

# 2.What measures do you think could be taken to achieve broader consensus in this field?
